# Antisense oligonucleotide-mediated exon skipping as a strategy to reduce proteolytic cleavage of ataxin-3

**DOI:** 10.1038/srep35200

**Published:** 2016-10-12

**Authors:** Lodewijk J. A. Toonen, Iris Schmidt, Martijn S. Luijsterburg, Haico van Attikum, Willeke M. C. van Roon-Mom

**Affiliations:** 1 Department of Human Genetics, Leiden University Medical Center, Albinusdreef 2, 2333ZA Leiden, The Netherlands

## Abstract

Spinocerebellar ataxia type-3 (SCA3) is a neurodegenerative disorder caused by a polyglutamine repeat expansion in the ataxin-3 protein. Cleavage of mutant ataxin-3 by proteolytic enzymes yields ataxin-3 fragments containing the polyglutamine stretch. These shorter ataxin-3 fragments are thought to be involved in SCA3 pathogenesis due to their increased cellular toxicity and their involvement in formation of the characteristic neuronal aggregates. As a strategy to prevent formation of toxic cleavage fragments, we investigated an antisense oligonucleotide-mediated modification of the ataxin-3 pre-mRNA through exon skipping of exon 8 and 9, resulting in the removal of a central 88 amino acid region of the ataxin-3 protein. This removed protein region contains several predicted cleavage sites and two ubiquitin-interacting motifs. In contrast to unmodified mutant ataxin-3, the internally truncated ataxin-3 protein did not give rise to potentially toxic cleavage fragments when incubated with caspases. *In vitro* experiments did not show cellular toxicity of the modified ataxin-3 protein. However, the modified protein was incapable of binding poly-ubiquitin chains, which may interfere with its normal deubiquitinating function. Low exon skipping efficiencies combined with reduction in important ataxin-3 protein functions suggest that skipping of exon 8 and 9 is not a viable therapeutic option for SCA3.

Spinocerebellar ataxia type 3 (SCA3), or Machado-Joseph disease, is a dominantly inherited neurodegenerative disorder with an onset around midlife and is characterized mainly by progressive ataxia affecting balance and gait^1^. SCA3 belongs to the polyglutamine (polyQ) family of disorders, which are all caused by expansion of a CAG repeat in the coding region of several different genes. In SCA3, the CAG repeat expansion is located in exon 10 of the *ATXN3* gene. Healthy individuals have a CAG repeat ranging from 10 to 51, whereas SCA3 patients have an expansion of 55 repeats or more^2^. The expanded CAG repeat is translated into a polyglutamine tract in the C-terminal region of the ataxin-3 protein.

Ataxin-3 is ubiquitously expressed, and though peripheral toxicity has been shown in recent years for polyQ disorders^3^, ataxin-3 toxicity occurs mainly in the brain. Neuronal loss is most prominent in cerebellum, pons and spinal cord^1^. Ataxin-3 is a deubiquitinating enzyme involved in the regulation of protein degradation. The C-terminally located ubiquitin-interacting motifs (UIMs) of ataxin-3 can bind ubiquitin chains and the N-terminal Josephin domain is able to cleave these bound chains^4^. The ataxin-3 isoform most abundantly expressed in brain contains a total of 3 UIMs^5^. Though the exact cellular mechanisms leading to pathogenesis have not been fully elucidated, the general consensus is that a gain of toxic function, rather than loss of wild-type function, is the driving force behind SCA3 disease progression pathology^6^.

A key role for the initiation of intracellular toxicity in polyglutamine disorders has been suggested to lie in the proteolytic cleavage of the mutant protein. Proteolytic cleavage can result in formation of shorter polyglutamine-containing protein fragments that are more toxic than the full-length protein and are prone to aggregation. Involvement of mutant ataxin-3 fragments has been suggested for several pathological processes such as: transcriptional deregulation, proteasomal and mitochondrial impairment, hindered axonal transport and impairment of autophagy^7^. Studies have shown that ataxin-3 can be cleaved by caspases^8^,^9^ and calpains^10^. These enzymes have several predicted cleavage motifs distributed throughout the ataxin-3 protein, and can hence generate protein fragments of varying sizes. C-terminal ataxin-3 fragments containing the polyQ expansion were detected in a SCA3 mouse model, as well as in patient brain areas most affected in SCA3, while they were not observed in unaffected regions or control brain^11^. Inhibition of calpain-mediated cleavage resulted in an alleviation of toxicity in neuroblastoma cells^12^ as well as in mouse brain, where reduced ataxin-3 aggregation and nuclear localisation were also observed^13^. These results imply that preventing proteolytic cleavage of the mutant ataxin-3 protein could reduce its toxicity. However, such general inhibition of proteolytic enzymes also affects many other pathways in which these enzymes are involved. A more specific approach to prevent generation of toxic polyQ fragments may therefore be to render the ataxin-3 protein more resistant to cleavage. One way to achieve this protein modification is through use of antisense oligonucleotides (AONs).

AONs are short synthetic strands of DNA or RNA that can interact with RNA transcripts. AONs can act through different mechanisms, depending on the chemical modifications and design. For instance, transcripts can be broken down through RNAse H-mediated mechanisms. Alternatively, by targeting AONs to mask specific splicing signals within exons or introns, exons can be hidden from the splicing machinery by steric hindrance of SR proteins^14^. In this manner, exons can be targeted for exclusion from the pre-mRNA, resulting in exon skipping^15^. When the RNA reading frame is maintained, a new internally truncated protein can be generated with this strategy. Use of AONs for disorders of the central nervous system has gained interest in recent years due to favourable distribution throughout the brain, widespread cellular uptake and the ability to specifically target single transcripts in monogenic neurodegenerative diseases^16^. Additionally, phase 1 clinical trial using intrathecally delivered AONs for amyotrophic lateral sclerosis and spinal muscular atrophy showed encouraging results on tolerability and distribution of AONs in the central nervous system^17^,^18^.

In the current study we induce skipping of *ATXN3* exons that encode the region of the protein containing several proteolytic cleavage sites. The aim is to form an internally truncated ataxin-3 protein that is less susceptible to proteolytic cleavage and the formation of toxic protein fragments. As an assessment of protein function, we determined the ubiquitin-binding capacity of the modified ataxin-3 protein and established the effect on DNA strand breaks.

## Results

### Combined exon 8 and 9 skipping results in a modified ataxin-3 Δ88aa protein lacking predicted cleavage sites

**Figure 1:**
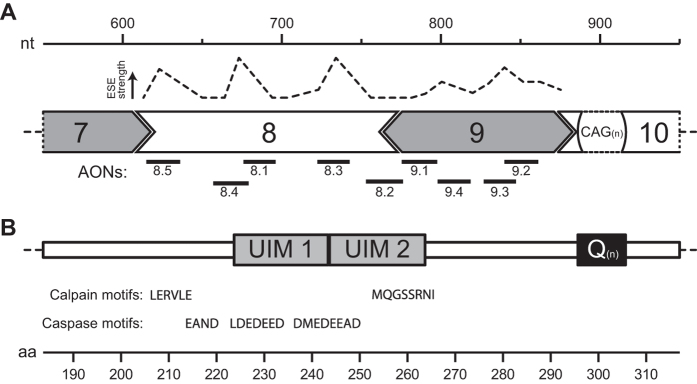
*ATXN3* exon structure and predicted cleavage motifs of ataxin-3. Based on Ensemble transcript ENST00000558190. (**A**) Depicted is the exonic splicing enhancer signal (ESE) strength as predicted by the human splicing finder. The shape of exons represents the influence on the RNA reading frame. Location of designed antisense oligonucleotides (AONs) against exon 8 and 9 to promote exon-skipping are drawn to scale. (**B**) The corresponding ataxin-3 protein region contains ubiquitin interacting motifs (UIMs) 1 and 2. Predicted cleavage sites N-terminal of the polyQ tract as detected for calpain^12^ and predicted for caspases^8^ are shown (not to scale). nt = nucleotides, aa = amino acids.

**Figure 2:**
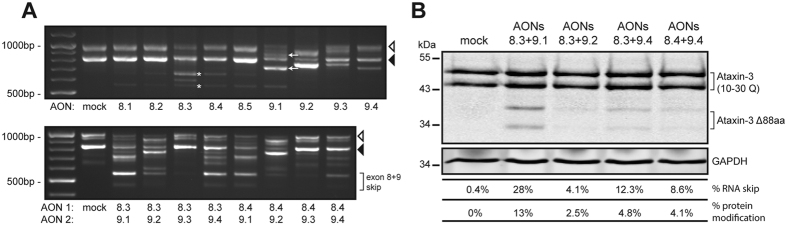
Ataxin-3 exon skipping in human fibroblasts. Control and SCA3 patient derived human fibroblasts were transfected with AONs complementary to exon 8 or 9 of *ATXN3*. (**A**) Screening of individual AONs for exon skipping activity at RNA level in SCA3 derived fibroblasts (white arrowhead = *ATXN3* 71 CAG, black arrowhead = *ATXN3* 10 CAG). For both the mutant and wild-type allele, the PCR product representing the single exon 8 skip is indicated with an asterix. Single exon 9 skip is indicated with an arrow. Combination of AONs for exon 8 and 9 were tested for simultaneous exon skipping (lower panel). (**B**) AON induced ataxin-3 protein modification. The most optimal AONs identified by the RNA screening were transfected in control fibroblasts and subjected to western blot analysis and anti-ataxin-3 probing. Percentage of exon skipping on RNA level as determined by qPCR and corresponding percentage of modification on protein level are indicated below.

Here, we devised a therapeutic strategy to remove exons 8 and 9 from ataxin-3 pre-mRNA, thereby eliminating 5 known or predicted proteolytic cleavage sites from ataxin-3 as depicted in Fig. 1. A total of 9 AONs were designed targeting *ATXN3* exon 8 and 9 (Table 1) and transfected in human fibroblasts in order to assess exon skipping efficiency. Using RT-PCR analysis with primers flanking the targeted exons, we determined that candidate AON 8.3 and 9.1 produced the most efficient skipping of exon 8 or 9, respectively (Fig. 2A). These AONs were subsequently tested in combination to induce simultaneous skipping of both exons. Sanger sequencing confirmed an in-frame *ATXN3* fragment where exon 7 was linked to exon 10. qPCR analysis showed that the most efficient AON combination, AON 8.3 and 9.1, induced up to 25% RNA modification (Fig. 2A) following a single transfection. Transfection of AONs 8.3 and 9.1 did not only result in a double skip of exon 8 and 9, but also in a single skip of exon 8 (167 bp). This is predicted to be a target for nonsense-mediated decay^19^ and therefore likely does not result in protein production. Single skipping of exon 9 (97 bp) was also observed for both alleles. This would lead to a reading-frame shift of exon 10 and potentially a novel protein product, but we did not detect this hypothetical protein in our western blot analysis (data not shown).

To determine the percentage of ataxin-3 protein modification after AON transfection in human fibroblasts, protein was isolated 48 hours after transfection. Following SDS-PAGE and western blotting, ataxin-3 protein was detected using an antibody specific to the C-terminal ataxin-3 region containing UIM3 (Fig. 2B). Two modified ataxin-3 proteins (ataxin-3 Δ88aa) of the expected size were detected at 34 and 40 kDa, corresponding to ataxin-3 derived from both alleles and lacking 88 amino acids (V204 to K291) with a maximum of 14% ataxin-3 protein modification. The C-terminal antibody was still capable of binding the modified protein, showing that there was no shift in the RNA reading frame after skipping of the two exons. Additionally, the modified protein was not detected with antibody 1H9, that recognizes ataxin-3 amino acids E214 to L233. This epitope is in the region of ataxin-3 that is not present after exon skipping again confirming exon skipping after AON transfection.

### Modified ataxin-3 Δ88aa protein is still cleaved by calpain-2

**Figure 3:**
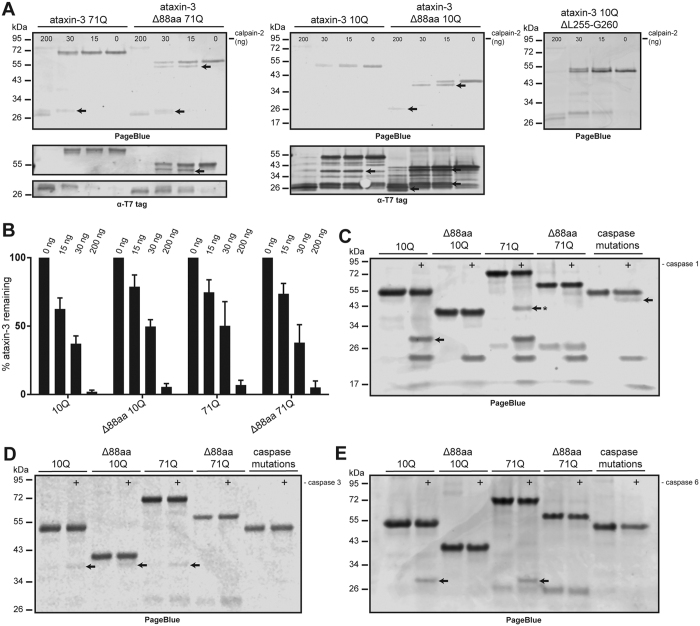
Modified ataxin-3 is cleaved less efficient by several caspases. (**A**) Formation of smaller ataxin-3 protein fragments are observed with increasing doses of calpain-2. Wild-type (10Q) and mutant (71Q) ataxin-3 were used either unmodified or lacking the targeted 88-amino acid region (Δ88aa). Ataxin-3 with a deletion of the previously identified calpain cleavage site (ΔL255-G260) was used as a control protein. There is a rapid decrease in full length protein and appearance of shorter protein fragments with increasing concentrations of calpain-2. After calpain-2 incubation, ataxin-3 Δ88aa with 10 and 71Q gives rise to a ~4 kDa smaller N-terminal fragment with PageBlue staining that is not observed for the wild-type protein. All fragments identified with Page Blue were positive when stained with an antibody against the N-terminal T7 tag (lower panels). Ataxin-3 ΔL255-G260 was cleaved similar to wild-type ataxin-3. (**B**) Quantification of uncleaved band intensity from (A) using Odyssey software. No significant differences were observed between rate of cleavage of ataxin-3 Δ88aa and the full length ataxin-3 protein variants. (**C**) Caspase-1 mediated cleavage of ataxin-3 resulted in a similar ~30 kDa N-terminal fragment for 10Q and 71Q ataxin-3 as indicated with the arrow. The corresponding C-terminal polyQ containing fragment (indicated with asterix) was detectable for ataxin-3 71Q. Upon removal of the 88aa neither cleavage fragment was observed for both 10Q and 71Q proteins, indicating the responsible caspases cleavage site is located in the targeted 88aa region. Bars depict mean and SE. (**D**) Overnight incubation with caspase-3 results in the appearance of a faint fragment of similar size for unmodified ataxin-3 10Q and 71Q, but also the proteins lacking 88aa. (**E**) Fragments of similar size for ataxin-3 10Q and 71Q were observed after incubation with caspase-6. Removal of 88aa prevented formation of this fragment, similar to ataxin-3 where the caspase cleavage sites were individually mutated.

A total of 3 caspase and 2 calpain cleavage motifs associated with polyQ toxicity are predicted to be located in the 88 amino acid region encoded by *ATXN3* exon 8 and 9 (Fig. 1). We hypothesized that the ataxin-3 Δ88aa protein lacking these 5 cleavage sites would be resistant to cleavage by caspases and calpains. In order to assess cleavage of ataxin-3 Δ88aa by caspases and calpains, we incubated purified recombinant ataxin-3 Δ88aa with caspases 1, 3 and 6 and calpain-2. Calpain-2 cleavage of ataxin-3 was more efficient compared to the caspases tested (Fig. 3A). Within 15 minutes of incubating 15 pmol ataxin-3 with calpain-2 at room temperature, all full-length protein was cleaved. Despite this efficient cleavage, few ataxin-3 fragments were detectable with PageBlue staining. This indicates that under these conditions calpain-2 does not generate a specific preferential cleavage fragment, or generates many fragments too small for detection. No difference in the rate of proteolytic cleavage between ataxin-3 Δ88aa and unmodified ataxin-3 was observed (Fig. 3B). Together, this shows that ataxin-3 Δ88aa, despite lacking predicted calpain-2 motifs ^209^LERVLE^214^ and ^257^MQGSSRNI^264^, is efficiently cleaved by calpain-2.

Upon overnight incubation with caspase-1, similar cleavage fragments were observed for wild-type ataxin-3 (10Q) and mutant ataxin-3 (71Q), with the most prominent fragment corresponding to a ~28 kDa N-terminal fragment of ataxin-3 (Fig. 3C). This finding was confirmed by western blotting with an antibody that recognizes the N-terminal T7 tag of the proteins. In case of ataxin-3 71Q, an additional ~42 kDa protein fragment was observed, likely corresponding to the C-terminal polyQ-containing fragment. Importantly, in the caspase-1 assay the corresponding ~28 and 42 kDa fragments were not observed for ataxin-3 Δ88aa with either 10 or 71 glutamines, indicating that the caspase-1 cleavage site was absent in the modified ataxin-3 protein.

The caspase-6 assay yielded similar results compared to caspase-1, with the N-terminal ~28 kDa fragment being the most prominent (Fig. 3D). The caspase-3 assay yielded different protein fragments. In this case, an N-terminal fragment of ~39 kDa was most evident (Fig. 3E). Probing with either a C-terminal antibody or 1C2, specific for the polyQ repeat, did not reveal the corresponding C-terminal protein fragment indicating the C-terminal fragment might be subject to further cleavage by caspase-3, rendering the fragment too small to be visible on the gel. However, due to inefficient cleavage by caspase-3 resulting only in small amounts of cleavage product, it is also possible that the C-terminal polyQ containing fragment was not abundant enough to be detected in these experiments.

### Absence of ataxin-3 UIMs 1 and 2 shows reduced binding of ubiquitin chains

**Figure 4:**
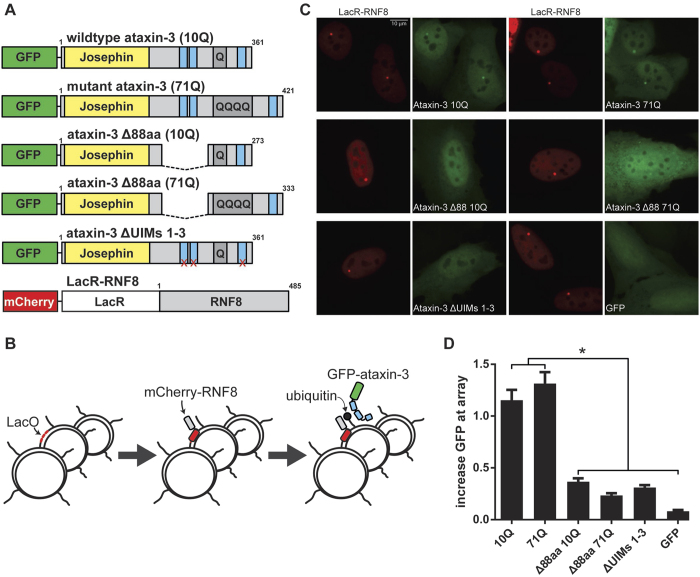
Modified ataxin-3 shows reduced binding of ubiquitin chains. (**A**) Schematic representation of ataxin-3 GFP-tagged proteins used for assessment of ubiquitin-binding with relevant functional domains depicted (not drawn to scale). For ataxin-3 ΔUIMs 1-3, the three UIMs were inactivated by point mutations. RFN8 ubiquitin ligase contained an N-terminal mCherry-LacR tag to enable visualization and targeting to the LacO array. (**B**) Representation of the histone ubiquitylation approach. A lacO repeat integrated into the genome of 2-6-3 U2OS cells allows targeting of the LacR-RNF8 fusion protein to the genomic LacO array, leading to localized chromatin ubiquitylation, which is bound by Ataxin-3. (**C**) Representative fluorescent images from transfected 2-6-3 U2OS cells expressing mCherry-LacR-RNF8 (red) and GFP-tagged Ataxin-3 proteins (green). Scale bar 10 μm (**D**) Quantification of ataxin-3 localisation at the LacO repeat. Increase in GFP signal at the site of mCherry signal signifies association of ataxin-3 with ubiquitin chains. Displayed values are the mean of 2 independent experiments in more than 50 cells per condition. *P ≤ 0.0001, bars depict mean and SE.

In order to establish whether ataxin-3 Δ88aa was able to bind ubiquitin chains through UIM3, we used a tethering approach to assess binding of ataxin-3 to locally induced ubiquitin moieties on chromatin^20^. To this end, 2-6-3 U2OS cells containing a stably integrated repeat array of the bacterial lactose operator (LacO) sequence^21^ were used. A fusion protein consisting of red-fluorescent protein mCherry, the RNF8 E3 ubiquitin ligase and the *Escherichia coli* lactose repressor (LacR) protein (mCherry-LacR-RNF8) was expressed in these cells, resulting in the tethering of the LacR-fusion protein to the LacO array and the local ubiquitylation of chromatin (Fig. 4B)^20^. Co-expression of GFP-tagged wild-type ataxin-3 (10Q) in these cells revealed recruitment of ataxin-3 (Fig. 4C,D) to ubiquitin conjugates at the LacO array. In contrast, ataxin-3 that contained point mutations in all 3 UIMs (L229A, L249A and L340A) was unable to associate with RNF8-mediated ubiquitin chains in this cell system, indicating that the recruitment of ataxin-3 to the array is mediated by its binding to poly-ubiquitin chains. Ataxin-3 containing the 71Q expansion associated with RNF8-mediated ubiquitin chains with similar efficiency as wild-type ataxin-3 (10Q), indicating that the expanded polyQ stretch does not affect the ubiquitin-binding activity of ataxin-3. However, ataxin-3 Δ88aa with either 10Q or 71Q, failed to associate with RNF8-induced ubiquitin chains, indicating that the Δ88aa region containing UIMs 1 and 2 is essential for ubiquitin binding.

### Ataxin-3 Δ88aa does not result in increased double stranded DNA breaks

**Figure 5:**
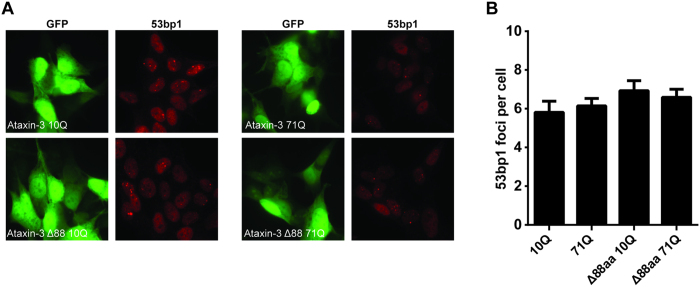
Expanded and modified ataxin-3 do not induce DNA strand breaks in neuroblasts. (**A**) SH-SY-5Y neuroblast cells transfected with GFP-ataxin-3 constructs were stained for the DNA double strand break marker 53BP1. (**B**) Quantification of 53BP1 foci in cells from A (>50 cells per condition), mean and SE depicted.

Recently it was shown that the expression of polyQ-expanded ataxin-3 can lead to genomic DNA damage in neuroblasts through inactivation of polynucleotide kinase 3′-phosphatase (PNKP)^22^. It was established that PNKP directly interacts with ataxin-3, but PNKP activity was specifically inhibited when ataxin-3 contained a 84 polyQ stretch. PNKP has a function in the repair of DNA double-strand breaks (DSBs)^23^, and its inactivation led to an increase in the accumulation of 53BP1 and γH2AX, which are key factors involved in the DSB response, into cytologically discernable foci containing DNA breaks^22^. In order to assess whether this DNA damage phenotype could be alleviated by removal of the 88aa, we expressed GFP tagged full-length ataxin-3 and ataxin-3 Δ88aa with either 10Q or 71Q in SH-SY-5Y neuroblasts and quantitatively assessed the number of 53BP1 foci in GFP-positive cells. The number of 53BP1 foci was not increased in cells expressing ataxin-3 71Q compared to ataxin-3 10Q, indicating a similar level of DSBs (Fig. 5B). Proper expression of the GFP-tagged ataxin-3 proteins was confirmed by western blot analysis (data not shown), showing that the expressed proteins contained both GFP and the polyQ stretch. For cells expressing the ataxin-3 Δ88aa with either 10Q or 71Q, no significant increase in 53BP1 foci was observed, indicating that there was no gain of toxic function for these proteins with regards to triggering genomic DNA damage.

## Discussion

Calpain-2 and caspase-mediated cleavage of ataxin-3 has been extensively investigated and is suggested to play a central role in polyQ mediated toxicity^8^,^9^,^10^,^12^,^13^,^24^. In the current study we confirmed the proteolytic cleavage of ataxin-3 by caspases and calpain-2, which is known to result in the formation of ataxin-3 fragments that can result in toxic and aggregation-prone expanded polyQ-containing fragments^25^. Indeed, *in vivo* experiments in which a mutant ataxin-3 fragment corresponding to a cleavage fragment around amino acids 221 or 260 was expressed in mice resulted in an aggravated neurologic phenotype^10^,^11^. AON-mediated removal of a central 88 amino acids region from ataxin-3 yielded a modified ataxin-3 protein predicted to be less prone to proteolytic cleavage and thus less toxic. When tested in proteolytic cleavage assays, the Δ88aa ataxin-3 protein did not prove more resistant to calpain-2 cleavage. However, Δ88aa ataxin-3 did prove less sensitive to cleavage by caspases 1, 3 and 6, and formation of the polyQ-containing fragment was abolished.

The Δ88aa ataxin-3 protein lacks 5 out of the 9 predicted caspase cleavage sites^8^. Pinpointing the exact calpain cleavage sites is more difficult because the mechanisms underlying substrate recognition by calpains are currently poorly understood. Based on experimental evidence, an important calpain cleavage site is thought to be located around amino acid 260 12. Due to their close upstream proximity to the polyQ repeat, both the calpain and caspase cleavage sites in the central 88 amino acids region of ataxin-3 are in a key position to generate a truncated polyQ containing fragment.

The calpain-2 cleavage assays performed here showed complete proteolysis of all tested ataxin-3 proteins at relatively low calpain concentrations, indicating that other cleavage motifs in the protein are used by calpain-2 when the central 88 amino acids are absent. We did not observe the toxic calpain-derived C-terminal fragment previously described in a cellular context^24^,^25^, but this could be due to methodological differences since we used purified proteins in our cleavage assay while Haacke and colleagues used a cell model. Using *in vitro* cleavage assays with purified caspases 1 and 6, we observed a different cleavage profile for ataxin-3 Δ88aa when compared to wild-type ataxin-3. Specifically, a prominent N-terminal fragment of approximately 30 kDa in size was no longer generated when incubated with caspase-1 and 6. Importantly, a 43 kDa polyQ-containing fragment was also abrogated in the case of the mutant ataxin-3 protein containing 71 glutamines. An ataxin-3 protein in which all predicted caspase cleavage sites were mutated also did not generate ataxin-3 cleavage fragments, indicating that the observed differences in proteolysis are likely the result of absence of cleavage motifs. In line with previous research, there was no increased caspase-mediated cleavage of expanded ataxin-3 compared to non-expanded ataxin-3. Whether caspase-mediated ataxin-3 cleavage is indeed a major contributor to SCA3 pathogenesis remains to be determined through future *in vivo* experiments.

*In silico* predictions using Cascleave^26^ software for Δ88aa ataxin-3 did not reveal generation of novel caspase cleavage motifs. In line with the increased resistance to caspase cleavage, we expected Δ88aa ataxin-3 to be less prone to calpain-2 cleavage as it lacks two key experimentally confirmed calpain-2 cleavage sites (Fig. 1A)^12^. It is therefore unexpected that the calpain-2 cleavage rates of Δ88aa ataxin-3 were identical to ataxin-3 13Q (Fig. 3B). This discrepancy between the two families of proteolytic enzymes can likely be explained by their mechanisms of substrate recognition. Substrate specificity of caspases has been extensively investigated, and is known to be highly dependent on specific peptide sequences^26^. In contrast to caspases, amino acid sequences around calpain cleavage sites are very diverse, and cleavage is likely also reliant on secondary and higher order structures^27^, making *in silico* prediction of calpain cleavage difficult. The comparable calpain-2 cleavage efficiency of Δ88aa ataxin-3 and wildtype ataxin-3 may hence be explained by previous observations, where amino acid substitutions at key positions in calpain substrates led to cleavage of adjacent sites^28^.

The Δ88aa ataxin-3 protein we describe here lacks UIMs 1 and 2, which are known to play a key role in the ubiquitin-binding function and specificity of ataxin-3^4^,^29^. We show that the remaining third UIM is unable to bind poly-ubiquitin chains using a cell system with localised chromatin ubiquitylation^20^ This observation is in line with a previously proposed functional model of ataxin-3, where the first 2 UIMs position ubiquitin chains to the catalytic Josephin domain in a linear fashion^30^. Additionally, as the valosin-containing protein (VCP) binding site is absent in Δ88aa ataxin-3, it may be useful to assess the effect of loss of VCP interaction in future experiments. VCP is described to be involved in ataxin-3 fibrillogenesis^27^, but also likely has a functional effect on the retrotranslocation of endoplasmic reticulum-associated degradation (ERAD) substrates^28^ as well as ataxin-3 activation^31^.

Recently it was shown that ataxin-3 interacts with the DNA end-processing enzyme PNKP. Interestingly, it was shown that wild-type ataxin-3 led to stimulation of PNKP, whereas expanded ataxin-3 led to inhibition^22^,^32^. PNKP has a crucial role in the initiation of DNA strand breaks through catalysing the restoration of 5′-phosphate and 3′-hydroxyl termini and interacting with other DNA repair proteins^23^,^33^. Hence, PNKP inactivation by expanded ataxin-3 may serve an important role in SCA3 pathogenesis, especially given the fact that the nervous system is particularly sensitive to DNA damage compared to other tissues^34^. It has been suggested that the PNKP inactivation by mutant ataxin-3 might in part be explained by its recruitment in polyQ aggregates^22^. Formation of polyQ aggregates, in turn, may be dependent on ataxin-3 cleavage and formation of short protein fragments containing the polyQ tract.

When expressing the Δ88aa ataxin-3 proteins, we did not observe an increase in the spontaneous formation of 53BP1 foci, which is a marker for DNA damage. However, we could not reproduce the previously reported ataxin-3-induced DNA damage phenotype in neuroblast cells^22^. One explanation for this discrepancy may lie in the fact that Gao and colleagues used an ataxin-3 protein with a slightly longer polyQ stretch (84Q) and used differentiated SY5Y neuroblast cells. Differentiation leads to alteration in cellular bioenergetics and oxidative stress response^35^ with increased oxidative vulnerability^36^ in these cells. It may therefore be useful to test the effect of ataxin-3 Δ88aa expression in differentiated neuroblasts in future experiments.

Our initial aim was to assess the therapeutic potential of AON-mediated removal of proteolytic cleavage sites of ataxin-3 for SCA3. Several advantages for the therapeutic use of AONs in the brain and central nervous system have been found over the years. These include: good distribution following infusion in the cerebrospinal fluid, excellent uptake in neurons and glial cells, high stability with half-lives of several months in the rodent brain and no adverse events in the clinical trials conducted thus far^18^,^37^. The AONs we describe in the current study likely do not provide a high enough level of ataxin-3 protein modification to be able to assess the influence of removal of these proteolytic cleavage sites from ataxin-3 on SCA3 neuropathology *in vivo*. Future studies could optimise the AON sequences against *ATXN3* exons 8 and 9 to provide a higher level of splicing modification prior to testing *in vivo*. AON potency may further benefit from more recent chemical modifications, which especially *in vivo* may yield significantly improved stability and potency (reviewed in^16^). Furthermore, the effect of the proposed protein modification on ataxin-3 functioning is an important consideration. Taken together, the fact that the AONs provide a low level of protein modification combined with the fact that ataxin-3 ubiquitin-binding function is reduced show that this approach is likely not an optimal therapeutic strategy for SCA3.

## Material and methods

All experiments described here are based on the ataxin-3 isoform RefSeq NM_004993.

### Antisense oligonucleotides

Uniformly modified single stranded 2′O-methyl phosphorothioate AONs of 20 to 23 nucleotides in length were designed according to previously described guidelines^38^ and are listed in Table 1. AONs were targeted against the most potent exonic splicing enhancer sites as predicted by the Human Splicing Finder software^39^. Care was taken to target a partially open secondary RNA structure of the relevant exon based on m-fold^40^. When possible, RNA hybridization strength, melting temperature, self-annealing and guanine-cytosine content were kept within previously described parameters^41^.

### Cell culture

Control or SCA3 patient-derived fibroblasts (GM02153 and GM06153 respectively) were obtained from Coriell Cell Repositories (Camden, USA). Fibroblasts were maintained in Minimal Essential Medium (MEM) (Gibco, Invitrogen, Carlsbad, USA), containing 15% fetal bovine serum (FBS) (Clontech, Palo Alto, USA), 1% Glutamax (Gibco), and 100 U/ml penicillin/streptomycin (Gibco).

Human U2OS 2-6-3 cells (kind gift from Dr. S. Janicki) containing 200 copies of a LacO (256×)/TetO (96×)‐containing cassette of ∼4 Mbp^21^ were cultured in Dulbecco’s Modified Eagle’s Medium (DMEM) (Gibco), supplemented with 10% FBS, 1% glutamax, and 100 U/ml penicillin/streptomycin. All cells were grown at 37 °C and 5% CO_2_.

Human neuroblast SH-SY5Y cells were maintained in a 1:1 mixture of DMEM and HAM’s F12 medium (Gibco) containing 15% FBS, 1% glutamax and 100 U/ml penicillin/streptomycin.

### Transfection and RNA analysis

AON transfections were performed as previously described^42^. In brief, Lipofectamine transfection reagent (Life Technologies, Paisley, UK) was diluted to 0.3% in MEM without supplements. A concentration of 200 nM of each AON was added to Optimem transfection medium (Life technologies) and incubated on cells for 4 hours, after which a 2–4 fold volume of growth medium containing 5% FBS was added. Plasmid transfections were transfected similarly to the AONs, but using 0.6% lipofectamine and 1–1.5 μg of plasmid DNA per 9.6 cm^2^ well. RNA was typically isolated 24 hours after transfection by trypsinizing (Life Technologies) cells and collecting the cell pellet. Cells were subsequently lysed and RNA was extracted using the Reliaprep RNA Cell Miniprep kit (Promega, Madison, USA) according to the manufacturer’s protocol. Between 200–400 ng RNA was used as input for cDNA synthesis using the Transcriptor First Strand cDNA Synthesis Kit (Roche, Mannheim, Germany). cDNA was synthesized according to manufacturer’s instructions using oligo-dT primers for 45 min at 50 °C. PCR to assess exon skipping was performed using the Expand High Fidelity PCR kit (Roche), with 1 μl of cDNA as template, 200 μM of each deoxynucleotide, 500 nM forward (exon 4) and reverse primer (exon 11) (see Table 2) (Eurogentec, Liege, Belgium), 1.7 units expand high-fidelity polymerase and PCR-grade water to a volume of 30 μl. PCR was performed with a 94 °C denaturation step of 2 min., followed by 36 cycles of 15 sec denaturation at 94 °C, 30 sec. annealing at 59 °C and 80 sec. of elongation at 72 °C, followed by a final extension step of 7 min. at 72 °C. To confirm exon skipping, Sanger sequencing was performed by extracting PCR products from gel using the NucleoSpin Gel & PCR Clean-up kit (Machery Nagel, Düren, Germany). The extracted PCR products were subsequently re-amplified, purified and submitted for Sanger sequencing (Macrogen, Amsterdam, the Netherlands).

Quantitative PCR analysis of exon skip efficiency was performed on exons of the *ATXN3* transcript using the SensiMix SYBR & Fluorescein Kit (Bioline, Taunton, USA), with 2 μl of 10x diluted cDNA, 100 nM forward and reverse primer and 4 μl 2x SensiMix SYBR & Fluorescein in a final reaction volume of 10 μl. The qPCR reaction was performed on the LightCycler 480 (Roche). Initial denaturation was 10 min. at 95 °C, followed by 40 cycles of 10 sec. denaturation at 95 °C, annealing at 60 °C for 30 sec and elongation at 72 °C for 20 sec. Reference genes used were: B-actin, hypoxanthine-guanine phosphoribosyltransferase (HPRT) and ribosomal protein L22 (Rpl22). All samples were ran in triplicate. Primers were designed with the help of Primer3 software^43^ and are listed in Table 2. Primer efficiencies were established using LinRegPCR v2014.0, using raw data amplification curves from the LightCycler software as input. After baseline correction, transcript level expression values (N0) were calculated and corrected for reference gene expression as described before^44^.

### Protein isolation and Western blotting

Fibroblasts were harvested 48 hours after AON transfection using 0.5% tripsin/EDTA solution and pelleted by centrifugation. After washing twice, cells were resuspended in lysis buffer^42^ supplemented with cOmplete EDTA-free protease inhibitors (Roche). Cells were disrupted by sonicating three times at an amplitude of 60 hz for 5 sec., followed by 30 minutes incubation in a head-over-head rotor at 4 °C. Protein concentrations were determined using the bicinchoninic acid kit (Thermo Fisher Scientific, Waltham, USA), with bovine serum albumin as a standard.

Protein samples were separated using 10% sodium dodecyl sulfate polyacrylamide gel electrophoresis (SDS-PAGE). Proteins were blotted onto a nitrocellulose membrane using the Transblot Turbo system (Bio-Rad, Hercules, USA), for 10 min. at 1.3 A. Blocking of membranes was performed using 5% low fat milk in tris buffered saline (TBS) for 1 hour. Blots were probed using an antibody specific for the C-terminal region of ataxin-3 (a kind gift from Dr. T. Schmidt)^45^ at a 1:1000 dilution. As loading control, GAPDH was used and detected using ab181602 (Abcam, Cambridge, UK) at 1:50.000 dilution. After washing, membranes were incubated for 1 hour with Odyssey secondary antibodies, goat-anti-mouse IRDye 680RD or goat-anti-rabbit IRDye 800CW (LI-COR Biosciences, Lincoln, USA) at a 1:5000 dilution. Bands were visualised using an Odyssey infrared imaging system (LI-COR). Protein bands were quantified with the Odyssey software version 3.0 using the integrated intensity method.

### Plasmids and mutations

Full length and exon 8 and 9 skipped products were obtained by performing PCR using primers (Table 2) specific for the full length *ATXN3* transcript. After extracting the PCR products of interest from agarose gel, the fragments were purified and ligated into the pGEM-T Easy vector (Promega), using the 5′-A overhangs on the PCR products. Constructs for mutant ataxin-3 were obtained by gene synthesis (Genscript, Piscataway, USA), using a mixture of 71 CAG and CAA codons instead of the pure CAG tract to improve stability during the sub cloning process. Plasmids were subsequently digested with NotI and inserted into the PspOMI digested PacGFP-C1 (Clontech, Mountain View, USA) vector for GFP transfection experiments, or into the NotI digested Pet28a vector (Merck Millipore, Billerica, USA) for recombinant protein production.

Mutations of UIMs and cleavage sites were introduced using the QuickChange II Site Directed Mutagenesis kit (Agilent Technologies, Waldbronn, Germany) according to manufacturer’s instructions using primers (Table 2) containing the desired mutation or deletion. Mutations of UIMs 1 and 2 have been described previously^46^. Predicted caspase sites encoded by exon 8 of ataxin-3^8^ were inactivated by replacing aspartic acid amino acids by aspargines. Altered aspartic acids were: D208, D217, D223, D225, D228, D241, D244 and D248. These 9 mutations were obtained by gBlock gene synthesis (Integrated DNA technologies, Coralville, USA) of the *ATXN3* cDNA sequence, which was subsequently amplified by PCR and sub-cloned into the Pet28a vector. mCherry-LacR-RNF8 was generated as described previously^20^. All constructs were Sanger sequenced to verify correctness.

### Recombinant protein production and purification

Hexa-HIS-tagged ataxin-3 proteins were produced using the Pet28a plasmid in a BL21 *E. coli* culture (New England BioLabs, Ipswich, USA). After 4 hours of protein production, bacteria were harvested by centrifugation and resuspended in BugBuster Plus (Merck Millipore) protein extraction reagent. HIS-tagged proteins were then bound using TALON slurry (Clontech) and finally dialysed overnight against PBS at 4 °C.

### Proteolytic cleavage assays

Calpain-2 cleavage assays were performed using 75 pmol of HIS-ataxin-3 protein with 15 to 200 ng calpain-2 (Calbiochem, Merck, Darmstadt, Germany) in reaction buffer containing 20 mM HEPES/KOH, pH 7.6, 10 mM KCl, 1.5 mM MgCl_2_, 1 mM dithiothreitol, and 1 mM CaCl_2_^12^, for a total volume of 50 μl. Caspase cleavage assays were performed with 50 pmol HIS-ataxin-3 protein with 200 U caspase 1 or caspase 6 (Enzo Life Sciences, Farmingdale, USA) in a buffer containing 50 mM Hepes, 50 mM NaCl, 10 mM EDTA, 10 mM DTT and 0.1% CHAPS. Cleavage reactions were incubated for 15 minutes at room temperature, after which reactions were stopped by addition of 4x Laemmli sample buffer (Bio-Rad) and heating to 100 °C for 5 min. Cleavage fragments were identified by 10% SDS-PAGE and PageBlue protein staining (Thermo-Fisher-Scientific) or Western blotting.

### RNF8-based chromatin tethering to assess ubiquitin binding of ATXN3 mutants

U2OS 2-6-3 cells^21^ containing the LacO repeat array were co-transfected with mCherry-LacR-RNF8^20^ in combination with GFP-tagged ataxin-3 constructs and grown on 18 mm glass cover slips (Thermo Fisher Scientific). Cells were fixed after 24 hours using 4% paraformaldehyde, and washed for 30 minutes using 0.2% Triton X-100 (Sigma-Aldrich, St. Louis, USA) in PBS. Cover slips were subsequently mounted on microscope slides using 0.002% 4′,6-diamidino-2-phenylindole (DAPI) in 1.3% 1,4-diazabicyclo[2.2.2]octane (DABCO) and stored overnight at 4 °C prior to imaging on a Leica DM-5500B fluorescent microscope (Leica Microsystems, Buffalo Grove, USA) at 63x magnification. Fluorescent images were obtained from 2 replicate transfections of a minimum of 50 cells positive for both mCherry-RNF8 and GFP-ataxin-3. The increase of GFP-ataxin-3 localization at the LacO array was determined by drawing a line region of interest across the array using LAS AF Lite software (Leica Microsystems), and subtracting green background fluorescence around the array from the maximum fluorescence intensity at the LacO array. The obtained value is reported as the increase of GFP signal at the array for the different GFP ataxin-3 constructs upon tethering of RNF8.

### Assessment DNA double-strand breaks

GFP-tagged ataxin-3 constructs were transfected in SH-SY5Y neuroblasts grown on collagen-coated 18 mm glass cover slips. 24 Hours after transfection, cells were fixed with 4% paraformaldehyde for 10 minutes and washed with PBS containing 0.5% bovine serum albumin (Sigma-Aldrich) and 0.15% glycine (Thermo Fisher Scientific) for 15 minutes. Slides were then incubated with rabbit anti-53BP1 at 1:1000 (Novus Biologicals, Littleton, USA) for 1.5 hours. After washing, detection was performed by incubation with anti-rabbit IgG Alexa 546 1:1000 (Invitrogen) for 1 hour and mounted with EverBrite hardset mounting medium containing DAPI (Biotium, Hayward, USA). Images of >50 cells were obtained the next day using an AxioImager M2 (Zeiss, Oberkochen, Germany) equipped with a 63x PLAN APO (1.4 NA) oil-immersion objective and an HXP 120 metal-halide lamp used for excitation. Foci were quantified using an ImageJ custom-made macro as described previously^47^. Only cells expressing the GFP proteins were included for analysis.

### Statistical analysis and calculations

Percentage of protein modification was calculated using the protein band mean Odyssey application software integrated intensity as follows: intensity modified protein/intensity all ataxin-3 bands * 100%. Ubiquitin-binding assays and calpain cleavage experiments were compared using 1 way ANOVA with Dunnet’s multiple comparisons test. A minimum of 50 cells from 2 replicates per construct were included for the ubiquitin-binding assays. Analysis of calpain-2 cleavage efficiency was derived from a total of 3 replicates. A *P* value of <0.05 was considered statistically significant.

## Additional Information

**How to cite this article**: Toonen, L. J. A. *et al.* Antisense oligonucleotide-mediated exon skipping as a strategy to reduce proteolytic cleavage of ataxin-3. *Sci. Rep.* **6**, 35200; doi: 10.1038/srep35200 (2016).

## Figures and Tables

**Table 1:** Antisense oligonucleotide sequences.

AON name	Sequence (5′ to 3′)
AON 8.1	GCCCUCUGCAAAUCCUCCUC
AON 8.2	CUUGCAUACUUAGCUGAAUAGCC
AON 8.3	CUGCUUCCUCAUCUUCCAUG
AON 8.4	CCUCAUCUUCGUCUAACAUUCC
AON 8.5	CACUCGUUCCAGGUCUGUUUU
AON 9.1	GAGAUAUGUUUCUGGAACUACC
AON 9.2	GCUUCUCGUCUCUUCCGAAGC
AON 9.3	CCGAAGCUCUUCUGAAGUAA
AON 9.4	CCUGAUGUCUGUGUCAUAUCU

**Table 2:** Primer sequences used for cloning, mutagenesis and (quantitative) RT-PCR.

Target gene	Primer name	Application	Sequence (5′ to 3′)
ATXN3	hATXN3_FL_Fw1	cloning	ATGGAGTCCATCTTCCACGA
ATXN3	hATXN3_FL_Rev	cloning	CGCATTGTTCCACTTTCCCA
ATXN3	hATXN3_L340A_Fw	mutagenesis	TATGAGTGAAGAAGACATGGCTCAGGCAGCTGTGACCATG
ATXN3	hATXN3_L340A_Rev	mutagenesis	CATGGTCACAGCTGCCTGAGCCATGTCTTCTTCACTCATA
ATXN3	hATXN3_del_L255-S260-Fw	mutagenesis	CTCCGCAGGGCTATTCAGTCCAGAAACATATCTCAA
ATXN3	hATXN3_del_L255-S260-Rev	mutagenesis	TTGAGATATGTTTCTGGACTGAATAGCCCTGCGGAG
ATXN3	hATXN3ex4Fw1	RT-PCR	GCCTTGAAAGTTTGGGGTTT
ATXN3	hATXN3ex11Rev1	RT-PCR	ACAGCTGCCTGAAGCATGTC
ATXN3	hATXN3-Qex3/4Fw1	qRT-PCR	GCCTTCTGGAAATATGGATGAC
ATXN3	hATXN3-Qex3/4Rev1	qRT-PCR	ATCGATCCTGAGCCTCTGATAC
ATXN3	hATXN3-Qex10/11Fw1	qRT-PCR	TCAGGACAGAGTTCACATCCA
ATXN3	hATXN3-Qex10/11Rev1	qRT-PCR	TTCAGGCAGCTGTGACCAT
ATXN3	hATXN3-Qex7/10Fw1	qRT-PCR	ATTGCGAAGCTGACCAACTC
ATXN3	hATXN3-Qex7/10Rev1	qRT-PCR	TTTTGCTGCTGTCTTTGCTC
ATXN3	hATXN3-Int11/11Fw1	qRT-PCR	AAATGTGGTTTTGTTTCCCAAC
ATXN3	hATXN3-Int11/11Rev1	qRT-PCR	ATGGTCACAGCTGCCTGAAG
ACTB	hACTBex4Fw1	qRT-PCR	AGCAAGCAGGAGTATGACGA
ACTB	hACTBex4Fw1	qRT-PCR	AGAAAGGGTGTAACGCAACTAA
HPRT1	hHPRT1ex3Fw1	qRT-PCR	GGGAGGCCATCACATTG
HPRT1	hHPRT1ex3Fw1	qRT-PCR	GTAATCCAGCAGGTCAGAAA
RPL22	hRPL22ex1Fw1	qRT-PCR	TCGCTCACCTCCCTTTCTAA
RPL22	hRPL22ex3Rev1	qRT-PCR	TCACGGTGATCTTGCTCTTG

Abbreviations: ACTB, beta actin; ATXN3, ataxin-3; HPRT1, hypoxanthine phosphoribosyltransferase 1; RPL22, ribosomal protein L22.
